# The first mitochondrial genome of scale insects (Hemiptera: Coccoidea)

**DOI:** 10.1080/23802359.2019.1622464

**Published:** 2019-07-10

**Authors:** Jun Deng, Congcong Lu, Xiaolei Huang

**Affiliations:** State Key Laboratory of Ecological Pest Control for Fujian and Taiwan Crops, College of Plant Protection, Fujian Agriculture and Forestry University, Fuzhou, China

**Keywords:** Mitochondrial genome, scale insects, *Ceroplastes japonicas*

## Abstract

Here, we report the first mitochondrial genome of scale insects sequenced from *Ceroplastes japonicus* (Hemiptera: Coccidae). The genome has a circular genome of 14,979 bp in length, with a high A + T content of 85.15%. Twelve protein-coding genes (excluding *atp8*), 13 tRNA, and 2 rRNA genes were detected and annotated using the MITOS web server. The absence of *atp8* and some tRNAs might indicate possible novel structures or loss of genes.

The scale insects (Hemiptera: Coccoidea) consist of more than 8300 species (García et al. 2016). Before the current work, no complete or partial (>5000 bp) mitochondrial genome from scale insects has been published in the GenBank (https://www.ncbi.nlm.nih.gov/), which makes it especially challenging and interesting to study and understand mtDNA evolution in Hemiptera.

*Ceroplastes japonicus* is a significant pest of ornamental plants in China (Deng et al. 2012). In this study, *C. japonicus* samples were collected in 2017 from *Gardenia jasminoides* in Ningbo of Zhejiang province, China. Specimen (voucher no. S2017-230) was deposited in the Insect Systematics and Diversity Lab at Fujian Agriculture and Forestry University, Fuzhou, China. Total genome DNA was extracted from 20 female adults of *C. japonicus* from the same twig using DNeasy Blood & Tissue Kit (Qiagen, Dalian, China). Raw data were generated on an Illumina HiSeq X Ten platform (Illumina, USA) resulting in 66 × 10^6^ paired-end 150 bp reads. The mitochondrial sequences were primarily assembled with IDBA-UD (Peng et al. 2012) and NovoPlasty software version 2.7.1 (Dierckxsens et al. 2017). A primer pair (c1-mt14781 5′-AGAAGCGGCTATTTTATGAGATTTGGAAG-3′ and c1-mt1385 5′-TGCTCATACGATGAATCCTAATACTCCGA-3′) was designed for both terminal ends, and the produce was sequenced by Sanger’s sequencing method. Finally, the draft mitochondrial genome was annotated using the MITOS web server (Bernt et al. 2013) with the protein prediction method of AL Arab et al. (2017), and deposited in GenBank under accession number MK847519.

The mitogenome of *C. japonicus* is a circular molecule of 14,979 bp in length and include 12 protein-coding genes (PCGs), 13 transfer RNAs (tRNAs), and 2 ribosomal RNAs (rRNAs). The nucleotide composition is significantly biased toward A + T (85.15%). The absence of *atp8* is observed in the current annotation. The *atp8* gene has a very short and poorly conserved sequence and tends to be missed by BLAST-based approaches (Al Arab et al. 2017). However, some cases of loss of *atp8* have been reported in nematodes, cnidarians, and flatworms (Okimoto et al. 1991; Beagley et al. 1996; Le et al. 2002). Meanwhile, all 13 tRNAs have no typical cloverleaf structure and are lacking the variable arm. Two tRNAs (trnN and trnS1) have the absence of the D-arm, and seven tRNAs (trnD, trnE, trnF, trnG, trnH, trnP, trnT) lack the T-arm. Our results indicate that some novel structural characters or loss of tRNA genes might occur in the mitogenome of *C. japonicus* even in other scale insects, which is needed to be further investigated based on more scale insect mitogenomes.

Twenty hemipteran species were selected to reconstruct a phylogeny with *C. japonicus* (Figure 1). Each PCG was aligned separately by MAFFT v7.0 (https://mafft.cbrc.jp/alignment/server/) (Katoh et al. 2017) and trimmed by Gblocks 9.1b (http://www.phylogeny.fr/one_task.cgi?task_type=gblocks) (Castresana 2000). Finally, we constructed a phylogenetic analysis involving 12 concatenated PCGs using IQ-TREE web server with default parameters (http://iqtree.cibiv.univie.ac.at/) (Nguyen et al. 2015; Trifinopoulos et al. 2016). The phylogenetic tree shows that Coccoidea represented by *C. japonicus* is the sister group of Aphidoidea and different clades have high support values.

**Figure 1.**
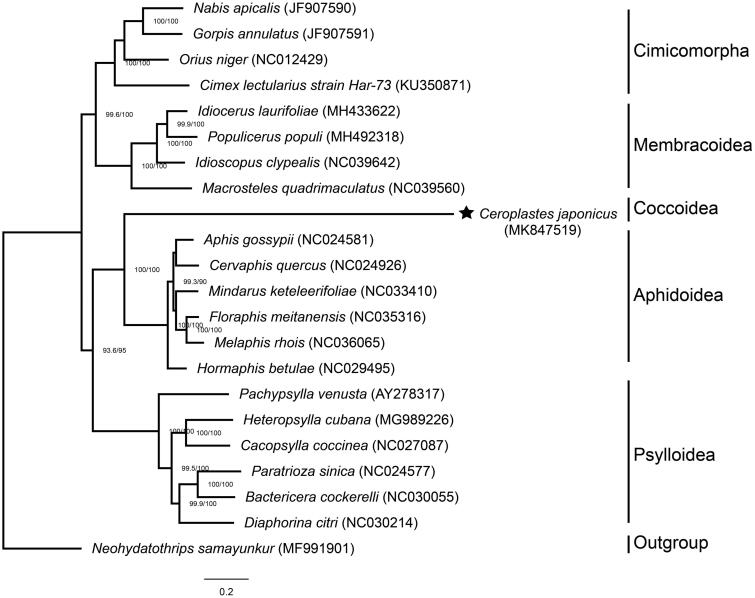
Maximum-likelihood phylogenetic tree of 21 Hemiptera species including *Ceroplastes japonicus* based on 7882 bp of 12 concatenated PCGs. Nodal numbers represent SH-aLRT support (left) and ultrafast bootstrap support (right) with 1000 bootstrap replicates. The numbers lower than 80 are not presented.
